# The training effects of a continuing education program on nurses’ knowledge and attitudes to palliative care: a cross sectional study

**DOI:** 10.1186/s12904-022-00953-0

**Published:** 2022-04-26

**Authors:** Xian Chen, Yuxi Zhang, Anne Arber, Xiaorong Huo, Jin Liu, Cuihua Sun, Ling Yuan, Xuemei Wang, Dan Wang, Jinfeng Wu, Junjie Du

**Affiliations:** 1 Jiangsu Nursing Association, Nanjing, 210008 China; 2 Department of Geriatrics, The First Affiliated Hospital of Nanjing Medical University, Nanjing, 210029 China; 3 Faculty of Health and Medical Sciences, The University of Surrey, Guildford, GU2 7XH UK; 4 Clinical Medicine Research Institution, The First Affiliated Hospital of Nanjing Medical University, Jiangsu Province Hospital, Nanjing, 210029 China; 5 Oncology Department, The Affiliated Hospital of Nanjing University Medical School, Nanjing, 210009 China; 6 Interventional Radiology Department, The First Affiliated Hospital of Nanjing Medical University, Jiangsu Province Hospital, Nanjing, 210029 China; 7 Oncology Department, The Affiliated Hospital of Xuzhou Medical University, Xuzhou, 221006 China; 8 Department of Geriatrics, The First Affiliated Hospital of Nanjing Medical University, Nanjing, 210029 China; 9 Department of Cardiovascular Surgery, The First Affiliated Hospital of Nanjing Medical University, Nanjing, 210029 China

**Keywords:** Attitude, Knowledge, Palliative care, Continuing education

## Abstract

**Background:**

Most nurses in China have not been trained to take care of end-of-life patients appropriately due to lack of educational resources and insufficient training. A palliative care program was launched by the Jiangsu Nursing Association (JNA training program) and to identify gaps in palliative care training. The main aim of this study was to evaluate the training effects of the JNA training program on nurses’ knowledge and attitudes to palliative care.

**Methods:**

A cross-sectional study was conducted with 10 048 registered nurses in all regions of Jiangsu. All participants completed an online questionnaire using the Chinese version of The Palliative Care Quiz for Nursing (PCQN-C) and the Frommelt Attitude Toward Care of the Dying scale (FATCOD-B-C). A propensity score matched analysis was performed between the nurses who had attended the JNA training program and whose who hadn’t.

**Results:**

The average score of PCQN-C among all nurses was 8.79, while the mean score of the FATCOD-B-C was 103.62. Those participants who attended the JNA training program had significantly better scores than those who did not. Propensity score matching analysis showed that the palliative care training program failed to improve nurses’ knowledge in psychosocial and spiritual care or their attitudes towards the necessity of family support although there was positive impact on other aspects of palliative care.

**Conclusions:**

Knowledge of palliative care among Chinese nurses remains low. Training programs may improve general knowledge and attitudes to palliative care. However, important aspects of knowledge such as communication skills, family support, and psychosocial aspects of care, are missing. These gaps should be filled in future palliative care training programs targeting nurses with oriental culture background.

**Supplementary Information:**

The online version contains supplementary material available at 10.1186/s12904-022-00953-0.

## Background

Palliative care aims to improve the quality of life for patients who have a serious or life-limiting disease [1]. It is now widely accepted that palliative care is a core component of the role of all health professionals who care for dying patients [2]. China is facing an unprecedented number of patients that require good quality palliative care because not only is China the most populous country in the world but also it has a rapid growth in the ageing population since entering the new century [3]. However, according to the 2015 Quality of Death Index [4], comparing countries’ hospitals and hospice environment, staffing numbers and skills, affordability of care, and quality of care, mainland China ranked 71^st^ out of 80 countries and regions.

This worrying concern about the quality of care of the dying in China aroused awareness and deep thinking of Chinese medical professionals as barriers that slowed the adoption of palliative care in China have been recognized [5–8]. For instance, the shortage of professional palliative care staff, especially nurses who are the major caregiver to patients at the end of life, is severe. In China, most nurses have not been trained to take care of end-of-life patients appropriately due to lack of educational resources and insufficient training [9–11]. A survey was conducted among 770 nurses from five Chinese provinces about their attitudes towards death and caring for the dying [12]. Most nurses in the study did not have a positive attitude towards care of the dying, associated with their attitudes towards death as well as their cultural backgrounds and religious beliefs. Another qualitative study showed that oncology nurses in China encountered serious dilemmas when delivering end-of-life care and struggled to provide psychological care and a lack of communication skills were identified [13].

China has been trying to improve the situation through various measures. The National Health Commission of China issued a series of documents to provide policy and administrative supports for hospice and palliative care in 2016 [14, 15]. A National Hospice Palliative Care Pilot Project has been launched since then which now takes in 29 provinces/municipalities covering 92 prefecture-level cities [15]. As part of this strategy, Jiangsu Nursing Association (JNA) has launched a continuing education program on palliative care since 2019 (referred to as “JNA training program” in this paper) as the backbone for pilot hospice care sites and nursing homes all over the province [16].

There has not been enough data available to evaluate the educational effects of the training program, not to mention the fact that fundamental information about palliative care knowledge and practice among Chinese nurses is still missing [17]. Therefore, this study compared Jiangsu nurses’ knowledge of palliative care and attitudes towards end-of-life care between those who had been enrolled in the JNA training program and those who had not to evaluate its training effects and to identify gaps in the training program of this kind that need to fill in the future.

## Methods

### Design and setting

A descriptive cross-sectional design was used in this study. The study employed a mobile phone app-based survey containing a three-part questionnaire (Wenjuanxing, www.wjx.cn). The data collection period was from 8^th^ October to 22^nd^ October 2020 (i.e., within a period of two weeks). The research was presented according to the STROBE checklist for cross-sectional research.

JNA distributed this anonymous, self-rated questionnaire to all nurses who attended the JNA training program in 2019 and other nursing members in Jiangsu via a WeChat applet and issued a notice to invite them to participate in the study. All responses from the nurses who had attended the JNA training program 2019 were included in the study. In order to represent the situation of nurses proportionally in the whole province, a regional-stratified sampling method was applied for responses from nurses who did not attend the training program. Registered nurses located in 13 prefecture-level cities in Jiangsu province were divided into 13 regions based on their geographic locations while the online questionnaire was distributed to all regions. All valid answers of the questionnaire from one region were included in the study when there were less than 1,000 responses in that region, otherwise 1,000 valid answers were randomly selected in case there were more than 1,000 responses in that region.

### Instruments

The online questionnaire consists of three parts: basic demographic and professional data, Chinese version of The Palliative Care Quiz for Nursing (PCQN-C), and Chinese version of the Frommelt Attitude Toward Care of the Dying scale (FATCOD-B-C).

### Demographic and professional data

The first part of the questionnaire included participants demographics, nursing profession and their experience in palliative care. Demographic characteristic of nurses included gender, age, marital status, and personal beliefs. Nursing professional background included hospital classification, working years, level of nursing job, current working department, whether is an oncology nursing specialist, whether is a palliative care nursing specialist. Palliative care experience, and whether attended the JNA training program 2019.

### The Palliative Care Quiz for Nursing (PCQN) instrument

The PCQN was originally designed by Ross et al. [18], and was translated into Chinese and validated by Zou [19]. The test–retest reliability was 0.782 and an internal consistency reliability of 0.758. The PCQN consisted of 20 questions with three possible responses including true, false and do not know. The PCQN contained three categories: (1) philosophy and principles of palliative care; (2) pain and symptom management; and (3) psychosocial and spiritual care [20]. The total score ranged from 0–20, the higher the score the better the knowledge of palliative care.

### The FATCOD-B-C instrument

The FATCOD-B was first developed by Formmelt to test nurses’ attitudes toward caring of the dying [21]. It was modified and transferred into Chinese version (the FATCOD-B-C) by Wang to assess nurses’ and nursing students’ attitude towards caring for end-of-life patients in Mainland China [22]. The FATCOD-B-C scale consisted of 29 items and it also classified into six subsets: Subset 1. attitude toward the interests of the dying person; Subset 2. attitude toward caring for the dying person; Subset 3. attitude toward the necessity of family support; Subset 4, attitude toward communication with the dying person; Subset 5. attitude toward fear of caring of dying person; Subset 6. attitude toward caring for the dying person’s families. It reported the construct validity of FATCOD-B-C was acceptable with Cronbach’s alpha coefficient 0.796 and the six subscales range form 0.610–0.863. The score from the 29 questions were added together to calculate a total FATCOD-B-C score with a possible range from 29–145, with the higher scores indicating more positive attitudes.

### Pilot study

Before data collection, a pilot study was conducted with 20 nurses from one local hospital to assess the clarity of the questionnaire and to evaluate the feasibility and clarity of the study. The questionnaire took on average 8 to 12 min to complete, and all the nurses in the pilot study found the questionnaire was clear and easy to understand. The data generated from the pilot study was not included in the data analysis.

### Ethical considerations

The ethical approval of this study was obtained from JNA and the First affiliated hospital of Nanjing Medical University (approval number: 2016-SRFA-076). All study participants were provided with informed consent electronically prior to participating in the study. The informed consent was at the beginning page of the survey which presented two options (Yes/No). Only subjects who chose “Yes” were guided to the questionnaire pages, and participants could quit the process at any time.

### Statistical analysis

To minimize confounding and selection biases, a propensity score matched analysis was performed between the nurses who attained the JNA training program 2019 and whose who did not. We estimated propensity scores using logistic regression modelling of baseline demographics and other covariates (hospital classification, working experience, level of nursing job, working department, oncology nursing specialist, palliative care nursing specialist, experience of caring for dying patients, and experience of discussing death with patients or their family members) which could have impact on the knowledge and attitude. We conducted propensity score-matching using 1:1 nearest neighbor matching without replacement with a caliper width of 0.1. Covariate distributions between trained nurses and untrained nurses were balanced after conditioning on the propensity score (Supplementary table 3).

Data were analyzed using IBM SPSS Version 23.0. Descriptive analysis was used to describe the general data. For count data, frequencies and percentages were used. Comparisons between trained nurses versus untrained nurses were conducted in both the overall cohort as well as the propensity score matched subset by using independent samples t-test for continuous variables and the results were presented as mean difference with 95% confidence interval of the difference. *P*-value < 0.05 indicated that a difference was statistically significant.

## Results

**Fig. 1:**
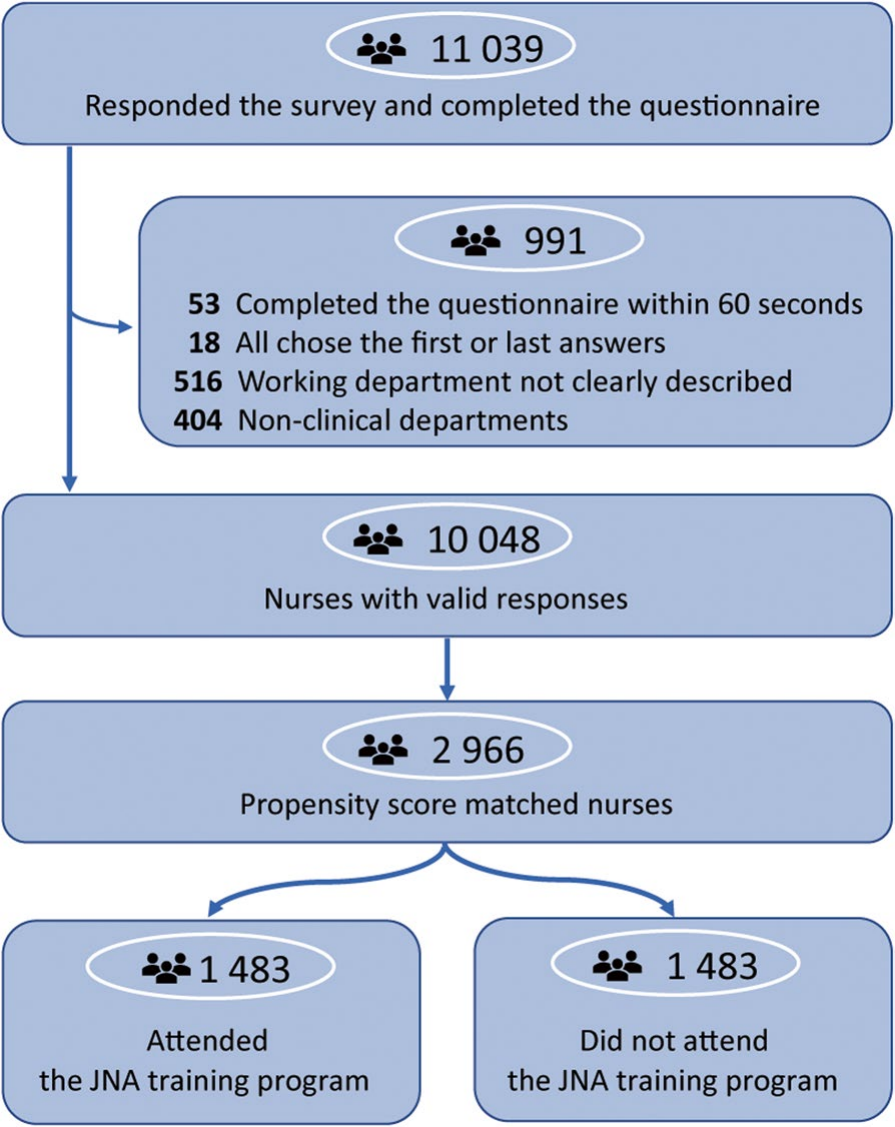
Study flowchart and propensity score matching of participants in the study

A total of 10,048 nurses were recruited to the study as indicated in Fig. 1. The demographic characteristics of all participants are shown in Table 1.

**Table 1:** Characteristics of the participants

Characteristic	Total number (percentage, %)
**Demographics**	
Gender	
Male	221 (2.10)
Female	9827 (97.90)
Age	
18–35	7539 (75.03)
36–50	2301 (22.90)
≥ 51	208 (2.07)
Marital status	
Single	3056 (30.41)
Married	6678 (66.46)
Divorced	157 (1.56)
Others	157 (1.56)
Personal beliefs	
Christianity	199 (1.98)
Buddhism	506 (5.04)
Muslim	10 (0.10)
Others	18 (0.18)
None	9315 (92.70)
**Nursing profession**	
Hospital classification	
Tertiary hospital	6860 (68.27)
Secondary hospital	2924 (29.10)
Primary hospital/Surgery	264 (2.63)
Working years	
≤ 5	3356 (33.40)
6–10	2964 (29.50)
11–15	1554 (15.47)
16–20	858 (8.54)
≥ 21	1316 (13.10)
Level of nursing job	
Junior level	6320 (62.90)
Medium level	2819 (28.06)
Senior level	909 (9.05)
Working department	
Department of oncology	1161 (11.55)
Department of geriatrics	429 (4.26)
Other departments of internal medicine	3393 (33.76)
Departments dealing with surgeries	2908 (28.94)
Pediatrics	434 (4.31)
ICU or Emergency department	1205 (11.99)
Outpatient department	518 (5.15)
Oncology nursing specialist	
Yes	147 (1.46)
No	9901 (98.54)
Palliative care nursing specialist	
Yes	65 (0.65)
No	9983 (93.35)
**Experience in palliative care**	
Experience of caring for dying patients^a^	
Yes	5256 (52.31)
No	4792 (47.69)
Experience of discussing death^b^	
Yes	4074 (40.56)
No	5974 (59.46)
Attended the JNA training program 2019	
Yes	1686 (16.78)
No	8362 (83.22)

*ICU* Intensive care unit, *JNA* Jiangsu Nursing Association

^a^A dying patient was defined in the present study as a patient with life-limiting illness and his/her life expectancy is 6 months or less

^b^This experience was defined as having discussed any topics of death with patients or with their family members

### The PCQN results: knowledge of nurses about palliative care

The mean knowledge of palliative care score was 8.79 (range 0–17, SD = 2.79) (Table 2). The distribution of nurses’ knowledge about palliative care on the PCQN scale was shown in Supplementary table 1. The mean knowledge for philosophy and principle of palliative care score was poor with 1.21 (range 0–4, SD = 0.96). The nurses’ knowledge regarding psychosocial and spiritual care in this study was poor with the mean score 0.5 (range 0–3, SD = 0.67). The nurses demonstrated better knowledge regarding pain and symptom control. The mean score was 7.08 (range 0–13, SD = 2.21).

**Table 2:** The knowledge of palliative care and attitudes toward care of the dying among all nurses

Questionnaires	Mean (SD)
Total PCQN score^a^	8.79 (2.79)
Category 1: Philosophy and principles	1.21 (0.96)
Category 2: Psychosocial aspects	0.50 (0.67)
Category 3: Control of pain and other symptoms	7.08 (2.21)
Total FATCOD-B-C score^b^	103.62 (11.07)
Subset 1. Attitude toward the interests of dying person	22.88 (3.36)
Subset 2. Attitude toward caring for the dying person	20.91 (4.01)
Subset 3. Attitude toward the necessity of family support	17.67 (1.64)
Subset 4. Attitude toward communication with dying person	13.18 (2.85)
Subset 5. Attitude toward fear of caring of dying person	9.30 (2.78)
Subset 6. Attitude toward caring for dying person’s families	16.42 (2.34)

*PCQN* Palliative Care Quiz for Nursing (Chinese version). FATCOD-B-C, the Frommelt Attitude Toward Care of the Dying scale (Chinese version)

^a^The full list of PCQN questions with all categories can be found in Supplementary table 1

^b^The full list of FATCOD-B-C with all subsets can be found in Supplementary table 2

### The FATCOD-B-C results: nurses’ attitudes towards care of the dying

The mean score of the FATCOD-B-C was 103.62 (range 65–145, SD = 11.07) (Table 2). We noticed that nurses had a poor attitude in communicating with the dying person. Especially, on question 8, with the majority identifying that they tend to be upset when the dying person is about to give up the hope (Supplementary table 2).

### Comparison between propensity score matched cohorts of trained and untrained nurses

We detected significant imbalances when comparing unmatched data of those attended the JNA training program 2019 and those who were untrained in terms of age, gender, marital status, hospital classification, working years, level of nursing job, working department, oncology nursing specialist, palliative care nursing specialist, experience of caring for dying patients and discussing death with patients or their family members. Following 1:1 propensity score matching, all covariates between both arms were well-balanced (Supplementary table 3).

In the overall unmatched cohort, the mean total PCQN and FATCOD-B-C scores of the trained nurses were significantly higher than those of untrained nurses. In the matched cohort, the result of PCQN score and FATCOD-B-C score also showed statistically significant differences between trained and untrained nurses. Also, the trained nurses had significantly higher scores on most categories of knowledge and subsets of attitude compared with untrained nurses both in the overall unmatched and matched cohorts. There was a statistic difference in the score of knowledge about psychosocial aspects between trained and not trained nurses before matching (0.54 ± 0.69 vs. 0.49 ± 0.66). However, such a difference between trained and not trained nurses disappeared after matching (0.54 ± 0.69 vs. 0.53 ± 0.65). (Table 3).

**Table 3:** Knowledge score and attitude score of nurses who attended or did not attend JNA training program 2019 before and after matching

	Before matching			After matching		
	Trained	Not trained	*P* value	Trained	Not trained	*P* value
No. (%) of nurses	1686 (16.78)	8362 (83.22)		1483 (50)	1483 (50)	
Total PCQN score, mean (SD)	9.85 (2.52)	8.57 (2.79)	0.00	9.66 (2.51)	8.95 (2.64)	0.00
Category 1. Philosophy and principles, mean (SD)	1.51 (0.98)	1.15 (0.95)	0.00	1.47 (0.98)	1.25 (0.95)	0.00
Category 2. Psychosocial aspects, mean (SD)	0.54 (0.69)	0.49 (0.66)	0.01	0.54 (0.69)	0.53 (0.65)	0.43
Category 3. Control of pain and other symptoms, mean (SD)	7.8 (1.99)	6.94 (2.22)	0.00	7.64 (1.97)	7.18 (2.07)	0.00
Total FATCOD-B-C, mean (SD)	108.17 (11.81)	102.71 (10.68)	0.00	107.54 (11.59)	104.11 (10.91)	0.00
Subset 1. Attitude toward the interests of the dying person, mean (SD)	23.84 (3.36)	22.69 (3.32)	0.00	23.66 (3.32)	23.12 (3.38)	0.00
Subset 2. Attitude toward caring for the dying person, mean (SD)	22.16 (4.04)	20.65 (3.93)	0.00	22.00 (4.04)	20.99 (3.94)	0.00
Subset 3. Attitude toward the necessity of family support, mean (SD)	21.29 (2.28)	20.87 (2.39)	0.00	21.26 (2.29)	21.00 (2.30)	0.00
Subset 4. Attitude toward communication with the dying person, mean (SD)	13.83 (3.03)	13.04 (2.79)	0.00	13.68 (2.98)	13.24 (2.82)	0.00
Subset 5. Attitude toward fear of caring of dying person, mean (SD)	9.91 (2.87)	9.18 (2.74)	0.00	9.87 (2.86)	9.29 (2.76)	0.00
Subset 6. Attitude toward caring for the dying person’s families, mean (SD)	17.14 (2.30)	16.28 (2.32)	0.00	17.06 (2.30)	16.47 (2.35)	0.00

*JNA* Jiangsu Nursing Association

**Fig. 2:**
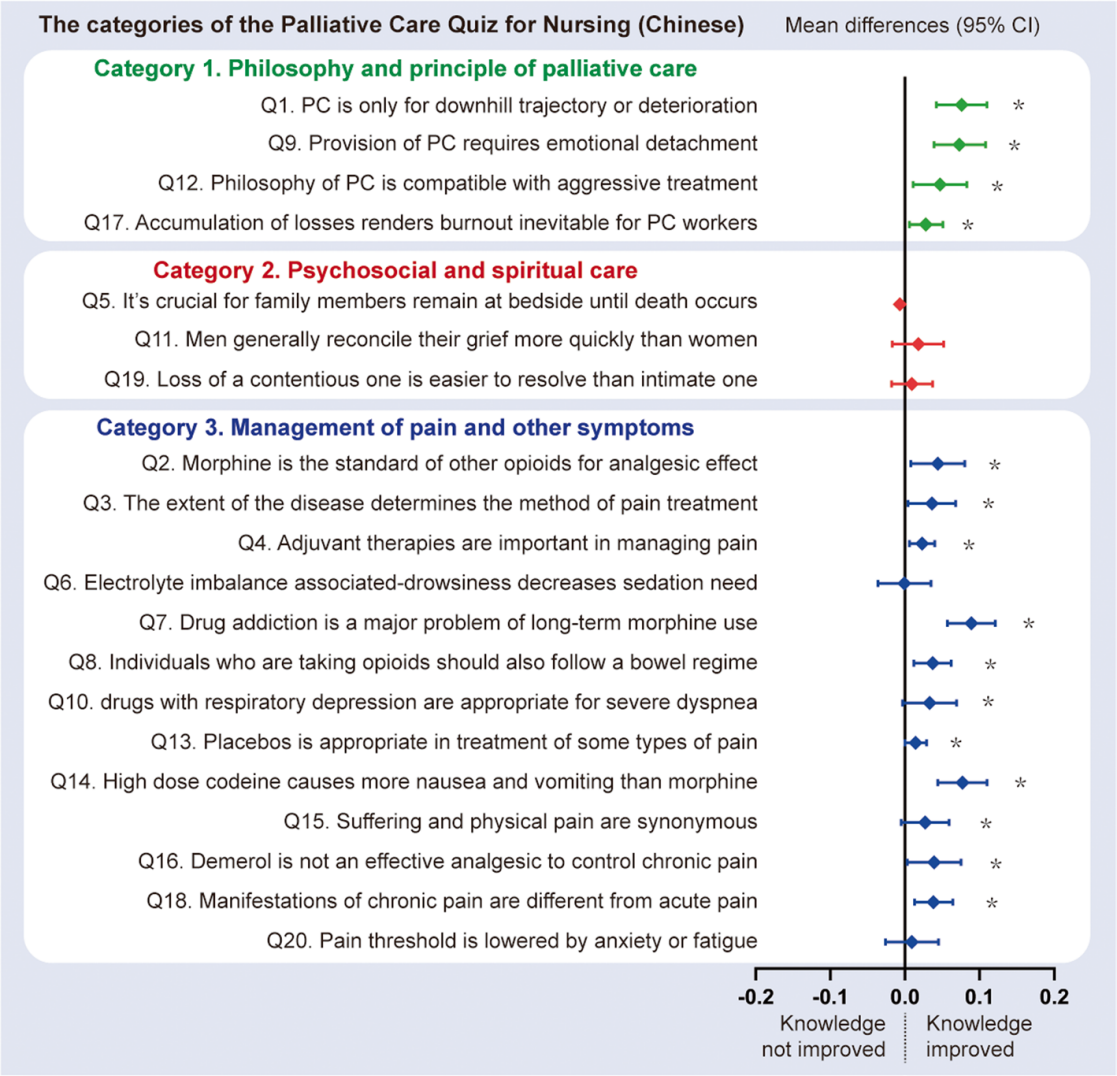
Mean difference of PCQN score between trained and untrained nurses after propensity score-matching. The questions of PCQN were listed and grouped into three categories with different colors at the left panel. The mean differences of the scores between those who attended the JNA training program and whose who did not attend after propensity score-matching were drawn with colored diamond dots with 95% confidence interval (95% CI) in line with each question on the right. The mean difference shifting towards right indicates the improvement of knowledge. * *P* value < 0.05. JNA, Jiangsu Nursing Association. PCQN, the Palliative Care Quiz for Nursing (Chinese version)

**Fig. 3:**
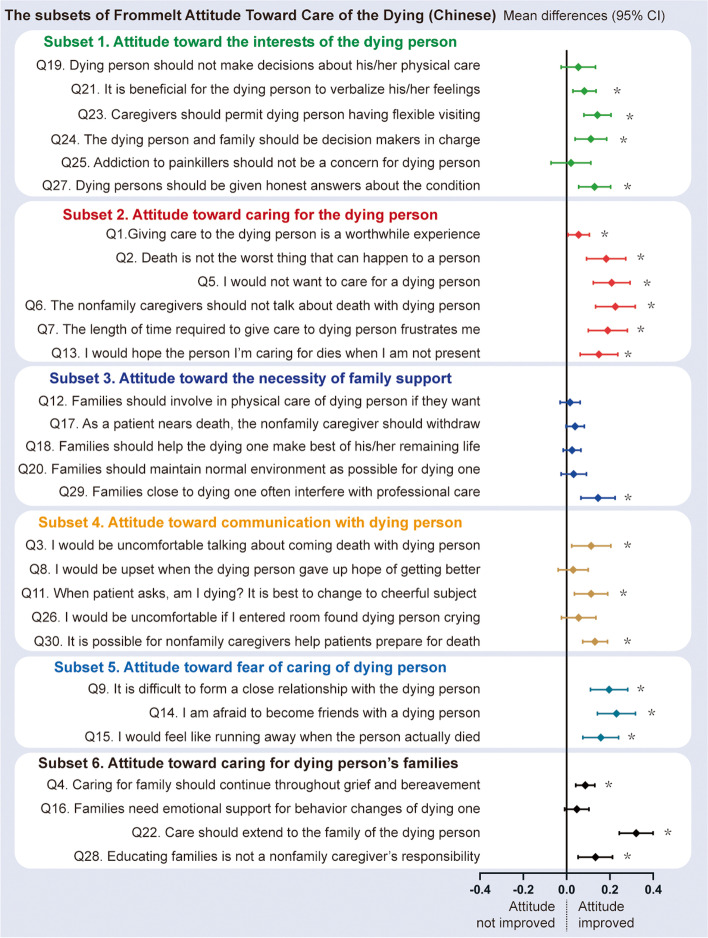
Mean difference of FATCOD-B-C score between trained and untrained nurses after propensity score-matching. The questions of FATCOD-B-C were listed and grouped into six subsets with different colors at the left panel. The mean differences of the scores between those who attended the JNA training program and whose who did not attend after propensity score-matching were drawn with colored diamond dots with 95% confidence interval (95% CI) in line with each question on the right. The mean difference shifting towards right indicates the improvement of attitude. * *P* value < 0.05. JNA, Jiangsu Nursing Association. FATCOD-B-C, Chinese version of the Frommelt Attitude Toward Care of the Dying

We further investigated the mean difference of PCQN and FATCOD-C-B in the matched cohort under the groups of categories or subsets item by item. Interestingly, all three items in the category of psychosocial and spiritual care of PCQN showed no difference between trained and untrained nurses while the mean differences of most other items were significantly different between two arms, which favours the improvement of palliative care knowledge of relevant categories (Fig. 2). Four out of five items in the subset of questions regarding the attitude toward the necessity of family support on the FATCOD-B-C showed no difference between trained and untrained nurses while the mean differences of most other items were significant between two arms, which favours the improvement of attitude toward relevant subsets (Fig. 3).

## Discussion

This cross-sectional survey recruited over 10,000 registered nurses all over Jiangsu province and revealed that their average level of knowledge and attitudes towards care for the dying were low while those who attended the JNA training program 2019 had significantly better knowledge and attitudes than those who did not. Propensity score matching analysis further showed that a training program may have positive impact on knowledge with learning and attitude gaining a higher score in most categories although it failed to demonstrate improvement in nurses’ knowledge in psychosocial and spiritual care or their attitude toward the necessity of family support.

Nurses are frequently exposed to dying patients and their families in their work and lack of knowledge of palliative and hospice care may lead to stress among nurses providing end of life care to those in need [23–25]. The average score of PCQN in the present study was 8.79 out of 20. This score is much lower than that of studies in other countries, such as the UK, the United States, Japan, and South Korea [26, 27]. This is in agreement with the Quality of Death Index (2015) which indicates that the palliative care in China is still behind most developed countries. Furthermore, our result is lower than that of other similar studies in China [28]. A plausible explanation lies in the distribution of participants in different studies. Previous Chinese studies recruited nurses from one or a few departments where palliative care is given frequently while nurses in our study come from all departments of hospitals where they may not often encounter dying patients. In fact, most nurses in our study are from departments other than oncology and geriatrics who may have less access to dying patients. Additionally, our results may also reflect another gap in how Chinese nurses are prepared to work with dying patients. All nurses, regardless which departments they are working at, are expected to understand the palliative care approach from their basic training these days. They are recommended to be exposed to education about palliative care during their initial training in school to understand what palliative care is [29]. However, there is a lack of initial preparation of Chinese nursing students as palliative care education and placement in hospice are still missing from our nursing schools. It really should be part of the curriculum by now and perhaps that is something to develop in the future.

Taking a close look at the PCQN results in our study, we found that most nurses seemed to perform better on the knowledge of pain management and other symptoms than on the knowledge of psychosocial and spiritual care or the philosophy and principle of palliative care. This reflects that Chinese nurses tend to pay more attention to patients’ physical symptoms while neglect their psychological need and social support for patients and their families as a result of medical education being centered on curative treatment of physical illness [30]. The lowest score was on Q5 (It’s crucial for family members remain at bedside until death occurs) which only 156 (1.6%) nurses in the study chose correctly. This result is similar to most studies conducted in China or in other Asian countries such as Korea. Lost in translation and cultural differences between the West and the East are used to explain this issue well [31] as it is traditional in Asia for family members, especially the son of the patient, to take a last look at the patient before he/she dies to do the filial respect to the patient regardless how difficult it might be.

Participants did report positive attitudes toward caring for the dying person’s families. In contrast, the attitude toward communication with the dying person was relatively poor, especially question 8: “I would be upset when the dying person I was caring for gave up hope of getting better,” which only scored 1.9. This may reflect a traditional view of death in China that Chinese people are often very afraid of death, as the old saying illustrates, “a living dog is better than a dead lion”. This attitude to death has huge impact on people’s behaviors when dealing with terminal illness for themselves and for their loved ones. To make it harder, speaking about death and dying is a taboo subject in traditional Chinese culture. It is challenging for nurses to tackle the communication aspects of care of the dying and to keep it culturally relevant and appropriate.

Although the fundamental knowledge and attitudes regarding palliative care among nurses were below expectation, the recently launched JNA training program was successful in improving knowledge and attitudes as the mean differences on examined items favour an improvement of knowledge and attitudes following education. These results were from a well-matched cohort in which most covariates that have impact on their knowledge or attitudes, other than attending the JNA training program, were fully considered and balanced.

The results also revealed some missing facets of curriculum provision of the training program as almost all items on the knowledge of psychosocial and spiritual care and attitude toward the necessity of family support showed no differences between trained and untrained nurses after matching. We reviewed the curriculum of the JNA training program 2019 which contained 18 keynote lectures and 8 onsite visiting and viewing and workshops. Only one lecture involved a topic of psychosocial support with one workshop introducing the model of internet plus for home care and family meeting. Therefore, it is not surprising to find these gaps in the training outcomes.

### Limitations

There are several limitations in the present study. First, we applied a regional-stratified sampling method to make sure that recruited surveys from each region are represented proportionally to the nursing population of Jiangsu province. However, convenient sampling was used within a region as we just collected all valid questionnaires in case there were less than 1 000 responses in that region which happened in eight regions. This might explain why most nurses were from tertiary hospitals while only a few were from primary hospitals in this study as city based-nurses are easier to approach via mobile phone app. Second, strictly speaking, the differences of knowledge and attitude between trained and untrained nurses cannot directly attribute to the JNA training program 2019 as this is a cross-sectional study which did not compare the outcomes of trained nurses before and after the training. However, we applied a propensity score matching to the cohort to balance other covariates that could have impact on their knowledge or attitude. Therefore, the results strongly implied the effects of the training program.

## Conclusions

This study has generated rich data to help nursing educators identify gaps and areas for improvement in the curricula of the palliative care training programs that are appropriate in oriental socio-cultural contexts. Generally, knowledge of palliative care among Chinese nurses remained low. Traditional training program may improve the general knowledge and attitude while some important facets, such as communication skills, family support, and the psychosocial aspects, are missing. These gaps should be filled in the curricula of future training programs targeting nurses with oriental culture background.

## Supplementary Information

**Additional file 1: Supplementary table 1.** Distribution of knowledge of palliative care based on their PCQN categories among all nurses. **Supplementary table 2**. The mean scores of FATCOD-B-C subsets among all nurses. **Supplementary table 3.** Comparison of characteristics of nurses who attended JNA training program 2019 and those who did not before and after propensity score matching.

## Data Availability

The datasets generated and/or analysed during the current study are not publicly available due the restriction from the Ethical Committee of the First affiliated hospital of Nanjing Medical University but are available from the corresponding author on reasonable request.

## Abbreviations

FATCOD-B-C: Chinese version of the Frommelt Attitude Toward Care of the Dying scale
JNA: Jiangsu Nursing Association
PCQN: The Palliative Care Quiz for Nursing
PCQN-C: Chinese version of The Palliative Care Quiz for Nursing
