# Supplementary material for: The training effects of a continuing education program on nurses’ knowledge and attitudes to palliative care: a cross sectional study
Source: BMC Palliat Care. 2022 Apr 26;21:56. doi: 10.1186/s12904-022-00953-0 (PMC9040326; doi:10.1186/s12904-022-00953-0)
Supplement: Supplementary file 1 — Additional file 1: Supplementary table 1. Distribution of knowledge of palliative care based on their PCQN categories among all nurses. Supplementary table 2. The mean scores of FATCOD-B-C subsets among all nurses. Supplementary table 3. Comparison of characteristics of nurses who attended JNA training program 2019 and those who did not before and after propensity score matching. [file 12904_2022_953_MOESM1_ESM.docx]

Supplementary table 1. Distribution of knowledge of palliative care based on their PCQN categories among all nurses

| Categories of PCQN^a^ | Correct, n (%) | Incorrect, n (%) |
| --- | --- | --- |
| **Category 1. Philosophy and principle of palliative care** |  |  |
| Q1. Palliative care is appropriate only in situations where there is evidence of a downhill trajectory or deterioration (False) | 3067 (30.52) | 6981 (69.48) |
| Q9. The provision of palliative care requires emotional detachment (False) | 3067 (30.52) | 6981 (69.48) |
| Q12. The philosophy of palliative care is compatible with that of aggressive treatment (True) | 5035 (50.11) | 5013 (49.89) |
| Q17. The accumulation of losses renders burnout inevitable for those who seek work in palliative care (False) | 964 (9.59) | 9084 (90.41) |
| **Category 2. Psychosocial and spiritual care** |  |  |
| Q5. It is crucial for family members to remain at the bedside until death occurs (False) | 156 (1.55) | 9892 (98.45) |
| Q11. Men generally reconcile their grief more quickly than women (False) | 3145 (31.30) | 6903 (68.70) |
| Q19. The loss of a distant or contentious relationship is easier to resolve than the loss of one that is close or intimate (False) | 1745 (17.37) | 8303 (82.63) |
| **Category 3. Management of pain and other symptoms** |  |  |
| Q2. Morphine is the standard used to compare the analgesic effect of other opioids (True) | 5117 (50.93) | 4931 (49.07) |
| Q3. The extent of the disease determines the method of pain treatment (False) | 2297 (22.86) | 7751 (77.14) |
| Q4. Adjuvant therapies are important in managing pain (True) | 9251 (92.07) | 797 (7.93) |
| Q6. During the last days of life, the drowsiness associated with electrolyte imbalance may decrease the need for sedation (True) | 5665 (56.38) | 4383 (43.62) |
| Q7. Drug addiction is a major problem when morphine is used on a long-term basis for the management of pain (False) | 1912 (19.03) | 8136 (80.97) |
| Q8. Individuals who are taking opioids should also follow a bowel regime (True) | 8273 (82.33) | 1775 (17.67) |
| Q10. During the terminal stages of an illness, drugs that can cause respiratory depression are appropriate for the treatment of severe dyspnea (True) | 5010 (49.86) | 5038 (50.14) |
| Q13. The use of placebos is appropriate in the treatment of some types of pain (False) | 409 (4.07) | 9639 (95.93) |
| Q14. In high doses, codeine causes more nausea and vomiting than morphine (True) | 6688 (66.57) | 3360 (33.43) |
| Q15. Suffering and physical pain are synonymous (False) | 6926 (68.93) | 3122 (31.08) |
| Q16. Demerol is not an effective analgesic in the control of chronic pain (True) | 5225 (52.00) | 4823 (48.00) |
| Q18. Manifestations of chronic pain are different from those of acute pain (True) | 8387 (83.47) | 1661 (16.53) |
| Q20. The pain threshold is lowered by anxiety or fatigue (True) | 6010 (59.81) | 4038 (40.19) |

^a^The quiz questions of palliative care for nursing were grouped based on their categories and were listed with the correct answers in the end.

Supplementary table 2. The mean scores of FATCOD-B-C subsets among all nurses

| FATCOD-B-C Subsets^a^ | Mean (SD) |
| --- | --- |
| **Subset 1. Attitude toward the interests of the dying person** | 22.88 (3.36) |
| Q19. The dying person should not be allowed to make decisions about his/her physical care. | 3.91 (1.12) |
| Q21. It is beneficial for the dying person to verbalize his/her feelings. | 4.30 (0.76) |
| Q23. Caregivers should permit dying persons to have flexible visiting schedules. | 4.11 (0.88) |
| Q24. The dying person and his/her family should be the decision makers in charge. | 3.85 (1.02) |
| Q25. Addiction to pain-relieving medication should not be a concern when caring for a dying person. | 3.55 (1.28) |
| Q27. Dying persons should be given honest answers about their condition. | 3.16 (1.02) |
| **Subset 2. Attitude toward caring for the dying person** | 20.91 (4.01) |
| Q1. Giving care to the dying person is a worthwhile experience. | 4.68 (0.71) |
| Q2. Death is not the worst thing that can happen to a person. | 3.78 (1.31) |
| Q5. I would not want to care for a dying person. | 3.50 (1.18) |
| Q6. The nonfamily caregivers should not be the ones to talk about death with the dying person | 3.25 (1.30) |
| Q7. The length of time required to give care to a dying person would frustrate me. | 2.80 (1.22) |
| Q13. I would hope the person I’m caring for dies when I am not present. | 2.89 (1.18) |
| **Subset 3. Attitude toward the necessity of family support** | 20.94 (2.38) |
| Q12. The family should be involved in the physical care of the dying person if they want to be. | 4.51 (0.66) |
| Q17. As a patient nears death, the nonfamily caregiver should withdraw his/her involvement with the patient. | 4.63 (0.61) |
| Q18. Families should be concerned about helping their dying member make the best of his/her remaining life. | 4.67 (0.59) |
| Q20. Families should maintain as normal an environment as possible for their dying member. | 4.27 (0.81) |
| Q29. Family members who stay close to a dying person often interfere with the professionals’ job with the patient. | 2.85 (1.08) |
| **Subset 4. Attitude toward communication with the dying person** | 13.18 (2.85) |
| Q3. I would be uncomfortable talking about impending death with the dying person. | 2.63 (1.23) |
| Q8. I would be upset when the dying person I was caring for gave up hope of getting better. | 1.93 (0.93) |
| Q11. When a patient asks am I dying? I think it is best to change the subject to something cheerful. | 2.26 (1.04) |
| Q26. I would be uncomfortable if I entered the room of a terminally ill person and found him/her crying. | 2.34 (1.09) |
| Q30. It is possible for nonfamily caregivers to help patients prepare for death. | 4.02 (0.82) |
| **Subset 6. Attitude toward fear of caring of dying person** | 9.30 (2.78) |
| Q9. It is difficult to form a close relationship with the dying person. | 2.94 (1.17) |
| Q14. I am afraid to become friends with a dying person. | 3.08 (1.21) |
| Q15. I would feel like running away when the person actually died. | 3.28 (1.14) |
| **Subset 6 Attitude toward caring for the dying person’s families** | 16.42 (2.34) |
| Q4. Caring for the patient’s family should continue throughout the period of grief and bereavement. | 4.62 (0.65) |
| Q16. Families need emotional support to accept the behavior changes of the dying person. | 4.30 (0.78) |
| Q22. Care should extend to the family of the dying person. | 3.62 (1.12) |
| Q28. Educating families about death and dying is not a nonfamily caregiver’s responsibility. | 3.88 (1.12) |

^a^Statements of attitude toward caring for the dying were grouped and listed based on their subsets.

Supplementary table 3. Comparison of characteristics of nurses who attended JNA training program 2019 and those who did not before and after propensity score matching

| Variable | No. (%) of nurses | |  | No. (%) of nurses | |  |
| --- | --- | --- | --- | --- | --- | --- |
|  | Before matching | | ASD | After matching | | ASD |
|  | Trained | Not trained |  | Trained | Not trained |  |
| Age, mean (SD), y | 33.29 (8.23) | 30.76 (7.47) | 0.31 | 32.72 (8.13) | 32.66 (8.11) | 0.03 |
| **Gender** |  |  |  |  |  |  |
| Male | 22 (0.01) | 199 (0.02) | 0.10 | 22 (0.01) | 20 (0.01) | 0.04 |
| Female | 1664(0.99) | 8163 (0.98) | 0.10 | 1461 (0.99) | 1463 (0.99) | 0.04 |
| **Marital status** |  |  |  |  |  |  |
| Single | 397 (0.24) | 2659 (0.32) | Ref. | 375 (0.25) | 392 (0.26) | Ref. |
| Married | 1233(0.73) | 5445 (0.65) | 0.18 | 1055 (0.71) | 1040 (0.70) | 0.01 |
| Divorced | 26 (0.02) | 131 (0.02) | 0.00 | 24(0.01) | 22 (0.01) | 0.01 |
| Others | 30 (0.02) | 127 (0.02) | 0.02 | 29(0.02) | 29(0.02) | 0.01 |
| **Personal beliefs** |  |  |  |  |  |  |
| Christianity | 28 (0.02) | 171 (0.02) | Ref. | 25 (0.17) | 23 (0.16) | Ref. |
| Buddhism | 103 (0.06) | 403 (0.05) | 0.05 | 88 (0.59) | 90 (0.61) | 0.02 |
| Muslim | 1 (0) | 9 (0) | 0.02 | 1 (0.01) | 1 (0.01) | 0.03 |
| Others | 5 (0) | 13 (0) | 0.03 | 4 (0.03) | 4 (0.03) | 0.00 |
| None | 1549(0.92) | 7766 (0.93) | 0.04 | 1365 (0.92) | 1365 (0.92) | 0.02 |
| **Hospital classification** |  |  |  |  |  |  |
| Tertiary hospital | 1323(0.79) | 5537 (0.66) | Ref. | 1146 (0.77) | 1124 (0.76) | Ref. |
| Secondary hospital | 308 (0.18) | 2616 (0.31) | 0.34 | 290 (0.19) | 312 (0.21) | 0.01 |
| Primary hospital/Surgery | 55 (0.03) | 209 (0.03) | 0.04 | 47 (0.03) | 47 (0.03) | -0.02 |
| **Working experience** |  |  |  |  |  |  |
| ≤5 years | 423 (0.25) | 2933 (0.35) | Ref. | 405 (0.27) | 417 (0.28) | Ref. |
| 6-10 years | 426 (0.25) | 2538 (0.3) | 0.12 | 387 (0.26) | 372 (0.25) | 0.01 |
| 11-15 years | 312 (0.19) | 1242 (0.15) | 0.09 | 264 (0.18) | 264 (0.17) | 0.01 |
| 16-20 years | 184 (0.11) | 674 (0.08) | 0.09 | 160 (0.11) | 165 (0.12) | 0.02 |
| ≥21 years | 341 (0.20) | 975 (0.12) | 0.21 | 267 (0.18) | 265 (0.18) | 0.03 |
| **Level of nursing job** |  |  |  |  |  |  |
| Junior level | 839 (0.5) | 5481 (0.66) | Ref. | 1146 (0.77) | 1124 (0.76) | Ref. |
| Medium level | 546 (0.32) | 2273 (0.27) | 0.11 | 290 (0.20) | 312 (0.21) | 0.01 |
| Senior level | 301 (0.18) | 608 (0.07) | 0.28 | 47 (0.32) | 47 (0.32) | 0.01 |
| **Working department** |  |  |  |  |  |  |
| Oncology department | 589 (34.9) | 572 (6.8) | Ref. | 397 (0.27) | 397 (0.27) | Ref. |
| Geriatrics department | 130 (7.7) | 299 (3.6) | 0.16 | 120 (0.08) | 120 (0.08) | 0.01 |
| Other internal medicine departments | 332 (19.7) | 3061 (36.6) | 0.43 | 332 (0.22) | 350 (0.24) | 0.01 |
| Departments dealing with surgeries | 390 (23.1) | 2518 (30.1) | 0.17 | 389 (0.26) | 366 (0.25) | 0.00 |
| Pediatrics | 44 (2.6) | 390 (4.7) | 0.13 | 44 (0.03) | 50 (0.03) | 0.01 |
| ICU or Emergency department | 126 (7.5) | 1079 (12.9) | 0.21 | 126 (0.09) | 134 (0.09) | 0.02 |
| Outpatient department | 75 (4.4) | 443 (5.3) | 0.04 | 75 (0.05) | 66 (0.04) | 0.05 |
| **Oncology nursing specialist** |  |  |  |  |  |  |
| Yes | 99 (0.06) | 48 (0.01) | Ref. | 25 (0.02) | 37 (0.02) | Ref. |
| No | 1587 (0.94) | 8314 (0.99) | 0.23 | 1458 (0.98) | 1446 (0.98) | 0.03 |
| **Palliative care Nursing specialist** |  |  |  |  |  |  |
| Yes | 41 (0.02) | 24 (0) | Ref. | 11 (0.07) | 17 (0.01) | Ref. |
| No | 1645 (0.98) | 8338 (1.00) | 0.14 | 1472 (0.93) | 1466 (0.99) | 0.03 |
| **Experience of caring for dying patients**^a^ | |  |  |  |  |  |
| Yes | 1299 (0.77) | 3957 (0.47) | Ref. | 1097 (0.74) | 1079 (0.73) | Ref. |
| No | 387 (0.23) | 4405 (0.53) | 0.71 | 386 (0.26) | 404 (0.27) | 0.02 |
| **Experience of discussing death**^b^ |  |  |  |  |  |  |
| Yes | 1153 (0.68) | 2921 (0.35) | Ref. | 957 (0.65) | 944 (0.64) | Ref. |
| No | 533 (0.32) | 5441 (0.65) | 0.72 | 526 (0.35) | 539 (0.36) | 0.02 |

Abbreviations: ASD, absolute standard different.

^a^A dying patient was defined in the present study as a patient with life-limiting illness and his/her life expectancy is 6 months or less.

^b^This experience was defined as having discussed any topics of death with patients or with their family members.
